# Formulation and Characterization of Muffins Enriched With Green Pea Powder: A Physical Study Using Response Surface Methodology

**DOI:** 10.1002/fsn3.70525

**Published:** 2025-06-27

**Authors:** Rabia Ilyas, Muhammad Nadeem, Nimrah Khan, Hafiz Muhammad Rizwan Abid, Colin J. Barrow, Nauman Khalid

**Affiliations:** ^1^ School of Food and Agricultural Sciences University of Management and Technology Lahore Pakistan; ^2^ College of Health Sciences Abu Dhabi University Abu Dhabi UAE; ^3^ Centre for Sustainable Bioproducts Deakin University Waurn Ponds Victoria Australia

**Keywords:** green pea powder, muffins, processed foods, response surface methodology

## Abstract

The study aimed to incorporate green pea powder (GPP) into muffins at an optimum level that reduces sugar content. Cooking time was optimized with the aim of maintaining the sensory quality of the muffins. The study employed a central composite design (CCD) and response surface methodology (RSM) to systematically optimize pea flour addition, sugar reduction, and cooking time optimization in muffins. The recipe refinement by RSM included muffins physical parameters (height, density, baking loss, volume, and mass) and sensory parameters (color, appearance, texture, taste, after taste, and overall acceptability). However, functional parameters like oil holding and water holding capacity were assessed and optimized for only the flour used for making muffins. RSM profiling optimized explanatory variables as 10% pea flour addition, 50% sugar reduction, and 22.7 min cooking. Based on this, the optimized final product surpassed predicted acceptability levels, particularly in taste (7.40), aftertaste (7.40), and overall liking (7.60). Seventeen runs were designed using various recipe combinations depending upon explanatory and dependent variables, and based on data profiling, an optimized recipe was developed, and point confirmation was done. Statistical analysis detected non‐significant results in height, density, and texture (*p* > 0.05 and *R* ^2^ < 80%). However, baking loss, volume, and mass exhibited significant results (*p* < 0.05 and *R* ^2^ 92%, 87%, and 96%), indicating better model fit. Results of this study indicate that pea protein can be incorporated into muffins while maintaining sensory properties.

## Graphical abstract

Green pea powder was added to muffins to make them a healthier snack. Response surface methodology was applied to optimize the pea flour addition and other conditions. Sensory properties were maintained in the optimized formulation.

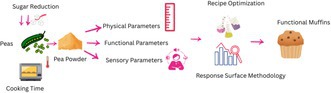

## 1 Introduction

Green pea ( *Pisum sativum* L.) is one of the world's oldest and most extensively utilized food crops (Kshetrimayum et al. 2022). Green pea are also an excellent source of essential amino acids, which contribute to their high‐quality proteins, although they lack sulfur‐containing amino acids. Countries consuming legumes, including peas, have less prevalence of colorectal cancers indicating that these are healthy protein rich ingredients (Kumar et al. 2019; Tibaldi et al. 2025).

Peas, utilized in various cuisines, include their grains and pods, which can confer a range of nutrients to developed foods. Garden pea ( *Pisum sativum* L.), an important winter vegetable, is processed seasonally. Peas and pea pods can be processed to powder for fortification for year‐round use, which is advantageous and also provides a solution to pea plant‐related waste (Gomes et al. 2021; Lamonaca et al. 2025; Tulbek et al. 2024).

Many food items include pea derivatives such as flour, starch, protein isolate, and pea fiber as valuable ingredients (Wu et al. 2023). A recent study reported that pea flour greatly boosted the protein level of bread and provided a well‐balanced amino acid profile when it was substituted for wheat flour (Ahmed et al. 2023; Kotsiou et al. 2025). Previously, researchers reported that dried green peas have a high lysine content, which makes them a great source of protein, especially when paired with foods high in sulfur amino acids but low in lysine (Boukid et al. 2021).

Fresh muffins are of the highest quality when elastic, well‐aerated, and have some spring (volume rise during baking) (Gülhan and Karaça 2023). Pea carbohydrates have a broad range of viscosities and temperatures compared to other cereals' starches. Peas contain glucose, galactose, arabinose, and other sugars, as well as larger polysaccharides, including stachyose and tetrasaccharide (Kumari and Deka 2021). As dry peas and other pulses are used in dhals, snacks, and soups, most research is being designed to fractionate pulse ingredients like starches, fibers, and proteins to diversify pulses (Li et al. 2019). Sigh and his colleagues reported that split peas have a lower glycemic index than green peas, thereby representing the possibility of using these ingredients to assist in controlling blood glucose and glycemic responses. However, results are inconsistent, which may be related to varying compositions as a result of processing method and cooking (Singh et al. 2021).

A statistical and mathematical method known as response surface methodology (RSM) is used to optimize process parameters for acceptable attributes of developed snacks (Hashemian et al. 2025; Lamidi et al. 2022). Research on baking gluten‐free bread was carried out previously by adding yellow pea powder, where processing parameters and finished bread quality parameters were optimized by applying RSM (Jeradechachai and Hall 2024). Nonetheless, little research has been done on recipe optimization by including legume powders, particularly pea powder, in cake flour and optimization using RSM for healthier snack options. The current research was designed to optimize the addition of pea powder and sugar along with optimal cooking time to produce organoleptically acceptable muffins by focusing on physical and sensory muffin attributes, which results from the functional parameters of muffin flour.

## 2 Materials and Methods

### 2.1 Chemicals and Reagents

The chemicals and reagents used in this research included acetic acid, chloroform, potassium iodide, sodium thiosulphate solution, and starch. All chemicals and materials were of analytical grade and were used as such.

### 2.2 Raw Materials Procurement

Raw green peas were purchased from the local market to make flour from green peas. Cake flour, cinnamon powder, cardamom, baking powder, egg, honey, and vanilla essence were also purchased from the local market.

### 2.3 Functional Properties of Muffin Flour

Flour should have good oil and water holding capacity, as it affects the functional characteristics of products incorporating the flour. The methods for determining oil and water holding capacity are described below.

#### 2.3.1 Water Holding Capacity (WHC)

To analyze the functional properties of flour, water holding capacities were determined using previously reported methods (Kim and Shin 2022) with minor modification. For WHC, 0.2 g samples were weighted and added into the centrifuge tube, then 1 mL distilled water was added and the sample mixed by vortex for 2 min. After resting at room temperature for 30 min, samples were centrifuged at 4000 rpm for 15 min. The supernatant was then removed, and the sediments were weighed.

#### 2.3.2 Oil Holding Capacity (OHC)

For oil holding capacity, 0.2 g of flour mixture homogenized with 1 mL melted butter was added to a pre‐weighed centrifuge tube and vortexed for 2 min using the method by Shi et al. (2020) with minor modifications. The WHC and OHC was calculated by taking the difference between the final weight (W_2_) and the initial weight (W_1_), as presented in Equation (2).

(1) $$\mathrm{WHC}\;\text{or}\;\mathrm{OHC}=\left[\left(W_{2}-W_{1}\right)÷\left(W_{0}\right)\right]\times 100$$

### 2.4 Preparation of Functional Muffins

Green peas were dried at 105°C for 24 h using a drying oven. After drying, green pea powder was made by grinding. This powder was then used to develop functional muffins. Table 1 shows actual and coded levels of explanatory variables applied for functional muffin preparation using 17 random runs.

**TABLE 1:** Actual and coded values for explanatory variables.

Run	Actual levels			Coded levels		
	Pea flour (%)	Sugar reduction (%)	Cooking time (min)	Pea flour (%)	Sugar reduction (%)	Cooking time (min)
1	10.00	50.00	20.00	−1.00	1.00	−1.00
2	30.00	30.00	23.00	1.00	−1.00	1.00
3	20.00	23.18	21.50	0.000	−1.68	0.000
4^a^	20.00	40.00	21.50	0.000	0.000	0.000
5	30.00	30.00	20.00	1.00	−1.00	−1.00
6	30.00	50.00	23.00	1.00	1.00	1.00
7	10.00	30.00	20.00	−1.00	−1.00	−1.00
8^a^	20.00	40.00	21.50	0.000	0.000	0.000
9	3.18	40.00	21.50	−1.68	0.000	0.000
10	10.00	50.00	23.00	−1.00	1.00	1.00
11	20.00	40.00	18.98	0.000	0.000	−1.68
12	30.00	50.00	20.00	1.00	1.00	−1.00
13	20.00	56.82	21.50	0.000	1.68	0.000
14	20.00	40.00	24.02	0.000	0.000	1.68
15	36.82	40.00	21.50	1.68	0.000	0.000
16^a^	20.00	40.00	21.50	0.000	0.000	0.000
17	10.00	30.00	23.00	−1.00	−1.00	1.00

^a^ Central.

### 2.5 Physical Characteristics

This study examined the physical properties of muffins, including their height, mass, volume, density, texture, and the baking loss incurred post‐baking.

#### 2.5.1 Height, Volume, Mass, and Density

Muffin height was measured by vertically halving the muffins and recording the height in cm. Muffin mass was measured by weighing. Volume was determined using the millet displacement technique, a method commonly used to measure bread volume. This technique involves displacing millet seeds or rapeseeds to determine the apparent volume of bread (Doddabematti Prakash et al. 2023). The specific volume (volume to mass ratio) was calculated by dividing the bread volume by its weight in grams. The density of the muffin was determined by dividing its mass by its volume.

#### 2.5.2 Baking Loss

The weight loss of muffins was measured using the AACC method on the electrical scale before and after baking (Heo et al. 2019). The baking loss was determined by subtracting the muffins' weight from the batter's weight and dividing this by the weight of the batter using Equation (1).

(2) $$\text{Baking loss}=\left(W1B-W2M\right)/\left(W1B\right)\times 100$$

#### 2.5.3 Texture

The texture of the pea powder in muffins was analyzed using the standard two‐bite method on a texture analyzer (Imada, Toyohashi, Japan). Muffins were placed on a plate of a texture analyzer using a cylindrical prob. mounted on the machine and operated at 4 mm per second. All hardness (N) values were measured in triplicate at room temperature by following the protocol by Jaiswal et al. (2024) with minor modification.

### 2.6 Sensory Characteristics

Sensory evaluation used the Likert scale (1 dislike extremely to 9 like extremely). Muffins were analyzed based on appearance, color, taste, aftertaste, overall acceptability, and texture (Gao et al. 2017). Sensory evaluation was carried out by a trained and semi‐trained panel, including 15 members aged between 25 and 40 years. The panel performed a sensory evaluation of muffins based on appearance, color, taste, aftertaste, overall acceptability, and texture according to their likes and dislikes. The prepared samples were coded with three‐digit numbers and were served randomly to every panelist to avoid biasness. Plain water was given to the panelists after every recipe of functional muffins.

### 2.7 Design of Experiment (DOE)

To optimize functional muffins, 17 runs based on explanatory variables were designed using CCD (central composite Design) with upper and lower levels, and evaluation was done based on dependent variables for physical, functional, and sensory parameters. Response surface models were created to predict dependent variable changes and optimize the desired outputs.

### 2.8 Statistical Analysis

Data were analyzed using Design Expert Software (Version 13). Various statistical parameters like *R* ^2^ (coefficient of determination) and ANOVA (analysis of variance) were checked to find the relevance and significance of regression models. Ranges of explanatory variables were prioritized in terms of equal, maximize, or minimize in the software and were analyzed accordingly. Point confirmation was carried out based on optimized parameters, and satisfactory outputs were reported.

### 2.9 Model Fitting for Muffin Evaluation

Pea flour, sugar reduction, and cooking time were explanatory variables. They were designed after preliminary trials and according to consumer's acceptance, whereas physical parameters (height, density, baking loss, volume, and texture), functional parameters (WHC and OHC), and sensory parameters (color, appearance, texture, taste, after taste and overall acceptability) were dependent variables. The generalized model encompassed dependent variables, intercepts, regression coefficients, quadratic effects, interaction effects of independent variables, and a model noise term or error. The equations reported in the results and discussion section against each variable have A, B, and C representing the pea flour, sugar reduction, and cooking time. AC, BC, and AB show the interaction between the explanatory variables, and A^2^, B^2^, and C^2^ show the quadratic effect of independent variables.

## 3 Results and Discussion

### 3.1 Effect of the Independent Variable on Functional Characteristics of Muffin Flour

**FIGURE 1:**
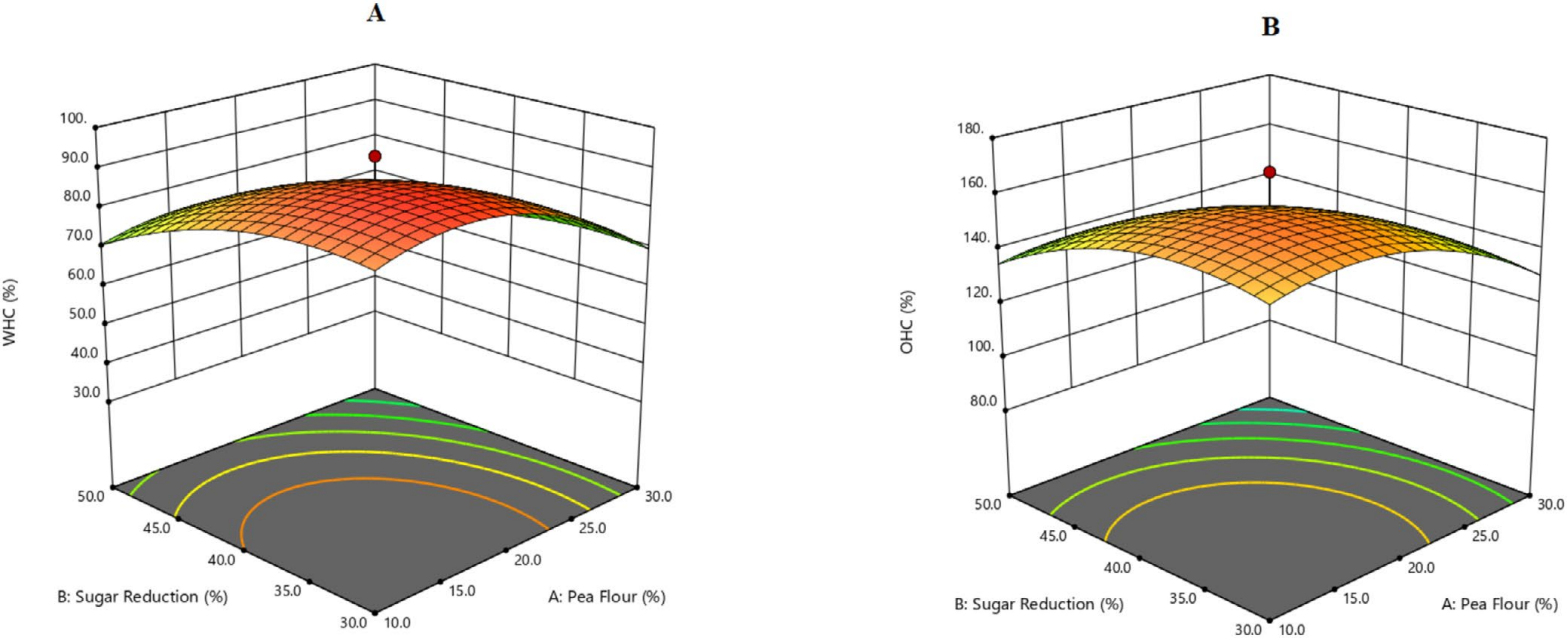
3‐D response surface graphs for the effect of explanatory variables, i.e., Pea Flour (%), Sugar Reduction (%), and Cooking Time (min) on Functional parameters of muffin flour: (A) WHC and (B) OHC.

A three‐dimensional figure (Figure 1A,B) was produced to show the visual effects of WHC and OHC based on the relationship between pea flour and sugar reduction, where the cooking time was constant at 21.50 min. Regression coefficients of applied polynomial equations are shown in Table 2, with WHC and OHC responses being detailed in Table S1. Explanatory variables reported significant effects (Table S2), with *R* ^2^ at 0.96 and 0.95 for WHC and OHC, respectively. A related study conducted by Khaleel et al. (2022) reported the addition of Kinnow peel powder of varying particle sizes for muffin quality and found WHC increased significantly, as the particle size increased from fine to coarse, while OHC increased as particle size reduced from coarse to fine. The increase of WHC and OHC in the current investigation is consistent with this study, indicating that the increase in WHC and OHC observed in the current study is associated with pea proteins, which entrap oil and water. Another study by Baldan et al. (2023) showed a non‐significant effect on OHC and a significant effect on WHC, for the quality of gluten‐free muffins incorporating grape pomace powder. These researchers reported that adding grape pomace powder prepared at 75°C conferred a high WHC due to the fiber matrix and high OHC due to protein traps. The final equation of functional parameters (WHC and OHC) of optimized functional muffins is presented in Equations (3) and (4).

(3) $$\begin{array}{c} Y1\left(\mathrm{WHC}\right)=9.33-0.4777A-0.4472B-0.3687C\\ -0.5910A^{2}-0.3559B^{2}-0.5122C^{2} \end{array}$$

(4) $$\begin{array}{c} Y2\left(\mathrm{OHC}\right)=12.47-0.5134A-0.4252B-0.4024C-0.0399\mathrm{AB}\\ -0.0470\mathrm{AC}+0.1126\mathrm{BC}-0.5398A^{2}-0.4559B^{2}-0.6006C \end{array}$$

**TABLE 2:** Regression coefficients of applied polynomial model for functional attributes of muffin flour.

Parameters		Intercept	A	B	C	AB	AC	BC	A^2^	B^2^	C^2^
Functional (flour)	**WHC**	**9.33**	**−0.478**	**−0.447**	**−0.369**	—	—	—	**−0.591**	**−0.356**	**−0.512**
	γ‐value		2.22E‐05	3.89E‐05	0.000188	—	—	—	8.01E‐06	0.000512	2.78E‐05
	**OHC**	**12.5**	**−0.513**	**−0.425**	**−0.402**	**−0.0399**	**−0.0470**	**0.113**	**−0.540**	**−0.456**	**−0.601**
	γ‐value		0.000735	0.00216	0.00291	0.744	0.701	0.371	0.000961	0.00249	0.000511

Bold values are statistically significant.

### 3.2 Effect of the Independent Variable on Physical Characteristics of Muffins

All physical responses are detailed in Table S3, and the regression coefficients of the applied models are shown in Table 3. Explanatory variables show the model's significance in variance analysis (Table S4).

**TABLE 3:** Regression coefficients of applied polynomial model for physical attributes of muffins.

Physical Parameters of Muffins	Intercept	A	B	C	AB	AC	BC	A^2^	B^2^	C^2^
Height	**2.04**	**0.00177**	**−0.00444**	**0.00121**	**0.00105**	**0.0191**	**0.00511**	**−0.00404**	**0.00746**	**0.0174**
γ‐value		0.805	0.540	0.865	0.911	0.0714	0.588	0.611	0.358	0.0556
Density	**0.914**	**0.0157**	**−0.00777**	**−0.00968**	**0.00597**	**0.00966**	**0.00120**	**0.0167**	**0.0178**	**0.00556**
γ‐value		0.0490	0.276	0.185	0.510	0.298	0.893	0.0551	0.0436	0.468
Baking Loss	**3.80**	**0.0454**	**0.0828**	**−0.180**	**0.305**	**0.172**	**−0.383**	**−0.0776**	**−0.224**	**−0.144**
γ‐value		0.425	0.167	0.0120	0.00335	0.0440	0.000939	0.230	0.00678	0.0448
Volume	**6.51**	**−0.121**	**0.0328**	**0.119**	**−0.115**	**−0.113**	**0.0797**	**0.103**	**0.122**	**0.188**
γ‐value		0.0213	0.450	0.0231	0.0690	0.0736	0.181	0.0567	0.0310	0.00426
Mass	**5.95**	**−0.00761**	**−0.0218**	**0.0459**	**−0.0717**	**−0.0372**	**0.0850**	**0.204**	**0.232**	**0.211**
γ‐value		0.741	0.356	0.0764	0.0419	0.238	0.0215	6.72E‐05	2.88E‐05	5.42E‐05
Texture	**4.15**	**−0.0108**	**0.0317**	**−0.0419**	**−0.0291**	**−0.0423**	**0.0272**	**−0.127**	**−0.300**	**−0.135**
γ‐value		0.954	0.864	0.821	0.904	0.861	0.911	0.538	0.170	0.513

*Note:* A = Pea Flour, B = Sugar reduction, C = Cooking Time and significance of model terms = γ‐value. Bold values are statistically significant.

#### 3.2.1 Height, Volume, Mass, and Density of Muffins

##### 3.2.1.1 Height

**FIGURE 2:**
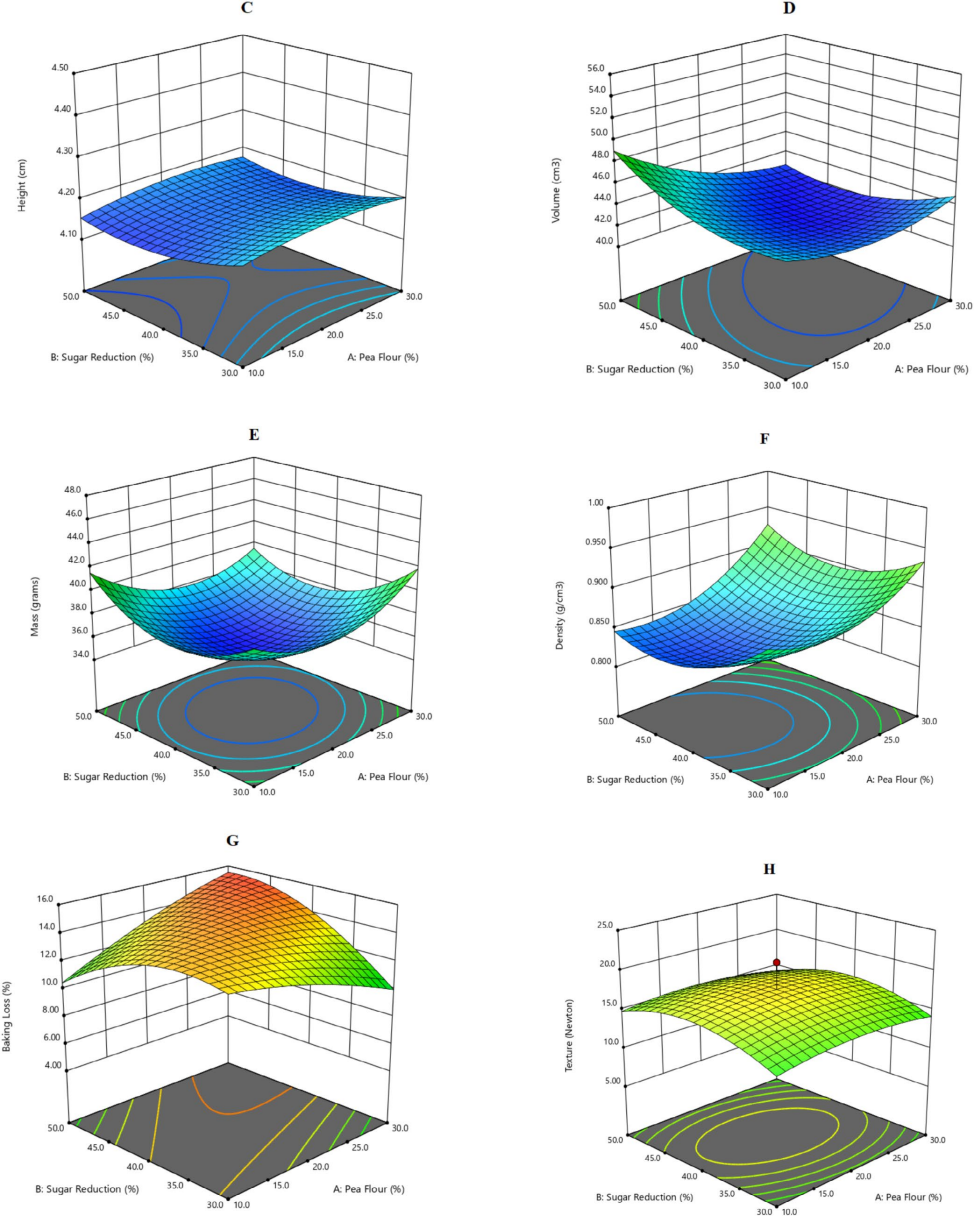
3‐D response surface graphs for the effect of explanatory variables, i.e., Pea Flour (%), Sugar Reduction (%) and Cooking Time (min) on physical parameters of functional muffins: (C) Height, (D) Volume, (E) Mass, (F) Density, (G) Baking Loss and (H) Texture.

The 3D surface graph (Figure 2C) shows the height of the muffin based on A‐pea flour (X1) and B‐sugar reduction (X2), with the cooking time kept at 21.50 min. The explanatory variable's effect on muffins' height is non‐significant (Table S4). However, observed small variations may be due to the incorporation of pea flour, and different levels of addition in muffins may impact height. The observed low coefficient of determination (*R* ^2^ = 0.64) shows less effect of explanatory variables on the height of muffins. Earlier, a study reported that microwave technology positively impacted muffin height. The fast heat transfer in microwave technology can affect height because it enhances the internal pressure of muffins and increases mass transfer from the inner layer to the product's surface. This can impact the height of muffins in different recipes (Rodríguez et al. 2022). A previous study reported that the muffin's hydrocolloid protein mixture can impact muffin height. The study showed that increased hydrocolloid protein increases muffins' height (Azmoon et al. 2021). The final equation for the height of the optimized recipe is presented in Equation (5).

(5) $$\begin{array}{c} Y3=2.04+0.0018A-0.0044B+0.0012C+0.0010\mathrm{AB}\\ +0.0191\mathrm{AC}+0.0051\mathrm{BC}-0.0040A^{2}+0.0075B^{2}+0.0174C^{2} \end{array}$$

##### 3.2.1.2 Volume

This three‐dimensional surface in Figure 2D shows the volume of muffins based on the relationship between pea flour and sugar reduction. Moreover, the explanatory variable shows a significant effect with a higher regression coefficient of determination, *R* ^2^ = 0.88 (Table S4). These findings indicate that pea flour composition, and in particular protein and carbohydrate composition, is important in enhancing the volume of muffins. This can be attributed to augmented water incorporation, which contributes to the development of a starch‐protein network and traps air during mixing, resulting in increased volume. In contrast, a study by Kutlu et al. (2024) reported decreased volume of the developed muffins as they incorporated back chickpea and/or aquafaba using four different treatments. This study reported decreased volume, which could be due to an inability of chickpea and aquafaba to retain air in batter, and also the fiber content of chickpea may have played its role in limiting water retention, impacting protein‐starch network formation and resulting in a reduction in volume. Similarly, Gomes and Aplevicz (2021) incorporated licuri flour in different bread variants. They concluded that the decrease in volume was due to a change in the viscoelastic properties of dough, which in turn produced a less soft product. The equation for the volume of optimized recipes is presented in Equation (6).

(6) $$\begin{array}{c} Y4=6.51-0.1212A+0.0328B+0.1189C-0.1151\mathrm{AB}\\ -0.1127\mathrm{AC}+0.0797\mathrm{BC}+0.1029A^{2}+0.1216B^{2}+0.1877C^{2} \end{array}$$

##### 3.2.1.3 Mass

A three‐dimensional surface (Figure 2E) was created by design expert software to show the mass of muffins based on the relationship between pea flour and sugar reduction. All three factors, including pea flour, sugar reduction, and cooking time, significantly affected the mass of muffins (Table S4), with a regression coefficient of determination (*R* ^2^ = 0.96). The current study incorporated muffins with green pea flour, which is rich in protein and may have the added benefit of high OHC and WHC, causing significant variation in mass. Jeong and Chung (2019) previously studied the effect of adding mung beans and cow along with waxy rice on gluten‐free muffins and observed an increase in the muffin's mass (weight) compared to the control, which is consistent with our findings. However, Ramya and Anitha (2020) added coconut flour (0%–25%) and honey as sweeteners to muffins and found significant variation among treatments in terms of mass. The final coded equation for the mass of optimized recipes is presented in Equation (7).

(7) $$\begin{array}{c} Y5=5.95-0.0076A-0.0218B+0.0459C-0.0717\mathrm{AB}\\ -0.0372\mathrm{AC}+0.0850\mathrm{BC}+0.2038A^{2}+0.2322B^{2}+0.2107C^{2} \end{array}$$

##### 3.2.1.4 Density

The 3D surface (Figure 2F) show the visual effects of the density of muffins based on the relationship or interaction between A‐Pea flour and B‐sugar reduction, with a consistent muffine cooking time of 21.50 min. Table S4 shows a non‐significant effect of treatment variables on the density of functional muffins (*R* ^2^ = 0.74). However, pea flour might not affect the density of muffins due to its good WHC, OHC, and adaptability to the batter. Recently, a study conducted by Meral (2022) showed the effect of mulberry fortification on the sensory, physical, and functional properties of gluten‐free muffins. Their batter density ranged from 0.87 to 0.91 g/mL. They reported that with the addition of 5%, 10%, and 15% mulberry extract, no change was observed in the density of batter, which is consistent with our results. In contrast, Román et al. (2015) suggested dough mixing, and the amount of aeration and emulsifier, reduce the density of the batter. Without an emulsifier, a more dense batter consistency is reported, and the reported density of cakes is in the range of 0.89–1.11 g/mL.

The equation for density for optimized recipes is presented in Equation (8).

(8) $$\begin{array}{c} Y6=0.9144+0.0157A-0.0078B\;0.0097C+0.0060\mathrm{AB}\\ +0.0097\mathrm{AC}+0.0012\mathrm{BC}+0.0167A^{2}+0.0178B^{2}+0.0056C \end{array}$$

#### 3.2.2 Baking Loss

The three‐dimensional graph in Figure 2G showed baking loss (%) based on A‐pea flour and B‐sugar reduction at a constant cooking time of 21.50 min. The strong agreement between experimental and predicted values exhibited a higher regression coefficient of determination (*R* ^2^ = 0.92), and the applied model reported a significant effect of treatment variables on baking loss (Table S4). Baking loss is imopacted by water holding capacity, where evaporation during baking has resulted in significant baking loss. A study conducted by Marchetti et al. (2018) reported that higher process yield resulted in bulkier product. Water loss during baking was the primary causes of weight loss. In this study they achieved higher process yields by substituting 20% or 30% of PEM (Pecan nut expeller meal) for wheat flour, resulting in less weight loss.

Similarly, Huang et al. (2021) studied the effects of water‐binding ingredients on baking loss and concluded that the increase of WHC has a positive influence on baking loss, as legume‐based flours have high WHC absorbed water which is trapped in the matrix of a baking product, thus decreasing weight loss. Their findings is consistent with our findings and illustrates the fundamental role of pea flour with high WHC in decreasing baking loss and enhancing the yield of the final baked item. The equation for baking loss of optimized recipes is presented in Equation (9).

(9) $$\begin{array}{c} Y7=3.80+0.0454A+0.0828B-0.1804C+0.3047\mathrm{AB}\\ +0.1717\mathrm{AC}-0.3829\mathrm{BC}-0.0776A^{2}-0.2237B^{2}-0.1439C^{2} \end{array}$$

#### 3.2.3 Texture of Muffins

The 3D surface (Figure 2H) exhibited the texture (hardness) of the muffin based on A‐pea flour (X1) and B‐sugar reduction (X2) at 21.50 min of baking. The addition of pea flour and sugar reduction did not affect the texture of the muffins, thereby reporting a non‐significant effect (Table S4) with a lower regression coefficient of determination *R* ^2^ = 0.27. The present study suggests the addition of pea flour, although it improved air trapping during better mixing, did not disturb the crumb hardness, thereby endorsing the addition of legume flour without compromising the texture. Previously, a study conducted by Olojede et al. (2020) reported the effect of cowpea and chickpea addition to sourdough breads and found a softer crumb texture, which was ascribed to better water retention and the emulsifying effect of added legume flour. Contrarily, another team of researchers found that adding kamut flour (traditional khorasan wheat) resulted in a harder texture than wheat flour but improved the nutritional profile of muffins. They attributed it to fewer air cells, which made the crumb compact and its texture (Lee et al. 2020). The final coded equation for the texture of optimized recipes is presented in Equation (8).

(10) $$\begin{array}{c} Y8=4.15-0.0108A+0.0317B-0.0419C-0.0291\mathrm{AB}\\ -0.0423\mathrm{AC}+0.0272\mathrm{BC}-0.1272A^{2}-0.3004B^{2}-0.1355C^{2} \end{array}$$

### 3.3 Effect of the Independent Variable on Sensory Characteristics of Muffins

**FIGURE 3:**
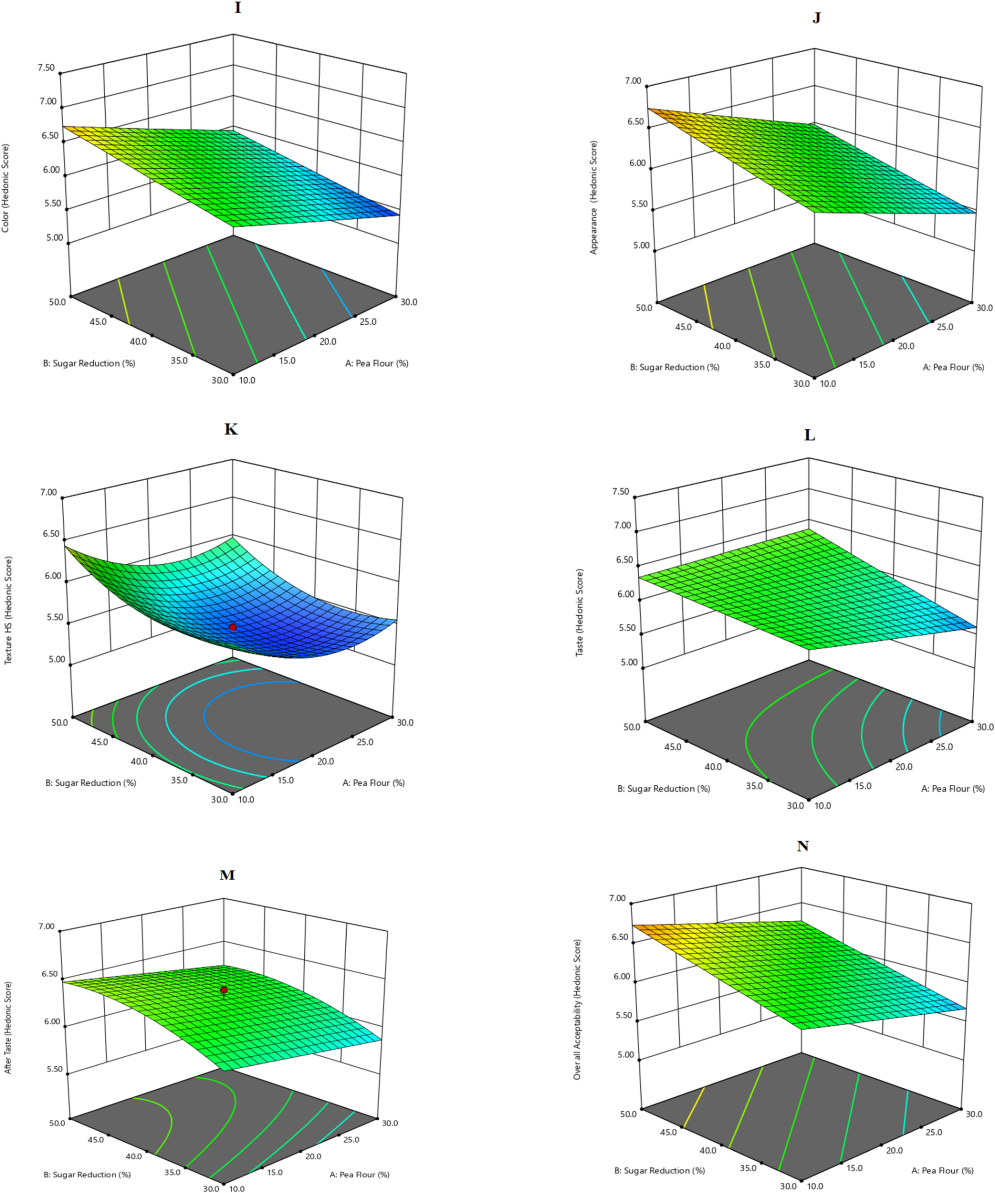
3‐D response surface graphs for the effect of explanatory variables, i.e., pea flour (%), sugar reduction (%), and cooking time (min) on sensory parameters of functional muffins: (I) Color, (J) appearance, (K) texture, (L) taste, (M) after taste, and (N) overall acceptability.

Before sensory evaluation, a questionnaire was formed to evaluate the recipes and determine the consumer's acceptance using the Hedonic scale. 3D response surfaces have been reported in Figure 3I–N, where pea flour, sugar reduction, and cooking time effects have been visualized. Nonetheless, the regression coefficients of applied polynomial equations on sensory parameters are shown in Table 4. However, numerical responses of all sensorial attributes have been reported in Table S5, whereas Table S6 depicts that all the sensory parameters had significant results, indicating the recipes had good acceptance among untrained judges. Regarding color, texture, appearance, taste, aftertaste, and overall acceptability, all the results reported a score (5–7), meaning recipes had good acceptance among consumers. These changes in sensorial traits of muffins are linked to added legumes like the chlorophyll of peas, which may affect the product's color and proteins and carbohydrates, which may affect the texture and taste. Pooja et al. (2022) incorporated pea flour into muffins and evaluated it using sensory parameters, including color, texture, taste, and overall acceptability. The results showed no significant difference in taste, but the muffins significantly differed in texture, color, and overall acceptability. Contemporarily, Kaur et al. (2022) developed muffins enriched with fiber using kimchi by‐products. The muffins were evaluated using different sensory parameters. This study depicted the contradictive results of sensory evaluation compared to the present study, which might be due to the biasness of the judges or people who might have different taste acceptance. As pea flour had no distinct taste and flavor under selected concentrations so its inclusion in muffins did not affect sensory in current research. Equations ((11), (12), (13), (14), (15)) presents the final equations for optimized functional muffins' sensory parameters.

(11) Color of functional muffins=2.46−0.0791A+0.0543B+0.0250C

(12) Appearance of functional muffins=2.47−0.0747A+0.0536B+0.0307C

(13) $$\begin{array}{c} \text{Texture of functional muffins}=2.34-0.0463A+0.0441B\\ +0.0540\mathrm{AC}+0.0604A^{2}+0.0508B^{2}+0.0446C^{2} \end{array}$$

(14) $$\begin{array}{c} \text{Aftertaste of functional muffins}=2.51-0.0247A\\ +0.0367B+0.0372C-0.0248B^{2} \end{array}$$

(15) $$\begin{array}{c} \text{Overall acceptability of functional muffins}=2.49-0.0466A\\ +0.0595B+0.0439C+0.0355\mathrm{AC} \end{array}$$

**TABLE 4:** Regression coefficients of applied polynomial model for sensory attributes of muffins.

Parameters		Intercept	A	B	C	AB	AC	BC	A^2^	B^2^	C^2^
Sensory (muffins)	Color	**2.46**	**−0.0791**	**0.0543**	**0.0250**	—	—	—	—	—	—
	γ‐value		0.00211	0.0209	0.249	—	—	—	—	—	—
	Appearance	**2.47**	**−0.0747**	**0.0536**	**0.0307**	—	—	—	—	—	—
	γ‐value		0.00121	0.0111	0.115	—	—	—	—	—	—
	Texture HS	**2.34**	**−0.0463**	**0.0441**	**0.0297**	—	**0.0540**	—	**0.0604**	**0.0508**	**0.0446**
	γ‐value		0.0139	0.0176	0.0825	—	0.0238	—	0.00570	0.0141	0.0259
	Taste	**2.48**	**−0.0300**	**0.0454**	**0.0425**	**0.0315**	**0.0453**	—	—	—	—
	γ‐value		0.0753	0.0128	0.0179	0.143	0.0445	—	—	—	—
	After Taste	**2.51**	**−0.0247**	**0.0367**	**0.0372**	—	—	—	—	**−0.0248**	—
	γ‐value		0.133	0.0341	0.0322	—	—	—	—	0.141	—
	Overall Acceptability	**2.49**	**−0.0466**	**0.0595**	**0.0439**	—	**0.0355**	—	—	—	—
	γ‐value		0.0107	0.00229	0.0148	—	0.104	—	—	—	—

*Note:* A = Pea Flour, B = Sugar reduction, C = Cooking Time and Significance of model terms = γ‐value. Bold values are statistically significant.

### 3.4 Point Confirmation of Optimized Recipe

Different parameters, including physical, functional, and sensory evaluation, were evaluated based on 17 standard runs. After analysis, an optimized recipe was reported with 10% pea flour addition, 50% sugar reduction, and 22.7 min cooking. Muffins were developed based on optimized parameters, and the relevant analysis results were confirmed in Table S7. All the experimental results were in the set ranges. Meanwhile, all sensory parameters exhibited higher values than upper limits, which indicates that the final recipe had more acceptability or likeness than the predicted results. A team of researchers (Heo et al. 2019) compared the acceptability of muffins made from various levels of dietary fiber, taste, and texture as the major factors affecting acceptability. Their optimized formulation also had high sensory scores similar to current research, where sensory parameters proved higher than predicted, indicating the acceptability of added pea powder muffins.

Later, Alemán‐Huerta et al. (2023) investigated whether incorporating kimchi by‐product powder into muffin formulations was possible and identified the perceived softness, flavor, and texture as major factors affecting product quality. The optimized formulations, similar to what was used in the present study, exhibited better consumer acceptance than anticipated, further supporting the postulate that functional and physical properties correlate with enhanced sensory characteristics. Similarly, Banu et al. (2023) reported that muffins produced with high‐protein ingredients (lupin, soy, and yeast proteins) tend to have higher sensory ratings than predicted, especially when the protein ingredients improve texture and flavor.

## 4 Conclusion

The current investigation applied response surface methodology using CCD to optimize incorporation of green pea flour into muffins, resulting in lower sugar levels and an optimized cooking time. The optimized method resulted in acceptable sensory quality of the muffins in addition to decreased sugar content. Optimized physical parameters represented numerical values as 4.43, 0.86, 8.08, 52.50, 45.10 and 5.50 for height (cm), density (g cm^−3^), baking loss (%), volume (cm^3^), mass (g) and texture (N), respectively. Similarly, functional parameters of flour explicated 67.21 and 138.40 for WHC (%) and OHC (%), respectively. Notably, the optimized recipe demonstrated favorable outcomes across sensory evaluations for muffins, highlighting its potential to cater to consumer preferences. Optimized sensory scores (Likert scale) were 7.47, 7.33, 7.13, 7.40, 7.40, and 7.60 for color, appearance, texture, taste, after taste, and overall acceptability, respectively. The current findings underscore the efficacy of CCD and RSM methodologies in fine‐tuning muffin recipes, in this case by incorporating pea flour, to meet sensory expectations while also decreasing muffin sugar levels. Future work could provide the nutritional profile for both the added green pea flour and the resulting muffin, to determine the nutritional improvement in muffin composition.

## Author Contributions

**Rabia Ilyas:** investigation (lead), writing – original draft (equal). **Muhammad Nadeem:** investigation (equal), methodology (equal), writing – review and editing (equal). **Nimrah Khan:** investigation (equal), methodology (equal), supervision (equal), writing – review and editing (equal). **Hafiz Muhammad Rizwan Abid:** formal analysis (equal), investigation (equal), methodology (equal), writing – review and editing (equal). **Colin J. Barrow:** project administration (equal), validation (equal), writing – review and editing (equal). **Nauman Khalid:** conceptualization (equal), funding acquisition (equal), investigation (equal), methodology (equal), project administration (equal), supervision (equal), writing – review and editing (equal).

## Ethics Statement

Ethical approval is not applicable in this paper.

## Conflicts of Interest

The authors declare no conflicts of interest.

## Supporting information


Tables S1–S7.

## Data Availability

All experimental data used to support the findings of this study are available from the corresponding author upon request.
