# Supplementary material for: Formulation and Characterization of Muffins Enriched With Green Pea Powder: A Physical Study Using Response Surface Methodology
Source: Food Sci Nutr. 2025 Jun 27;13(7):e70525. doi: 10.1002/fsn3.70525 (PMC12203390; doi:10.1002/fsn3.70525)
Supplement: Supplementary file 1 — Tables S1–S7. [file FSN3-13-e70525-s001.docx]

**Supplementary Data**

**Table S1: Experimental design using factors for responses of functional parameters of muffin flour**

| **Run** | **WHC (%)** | **OHC (%)** |
| --- | --- | --- |
| 1 | 69.33 | 129.67 |
| 2 | 53.67 | 101.67 |
| 3 | 83.67 | 144.33 |
| 4 | 84.33 | 153.67 |
| 5 | 70.00 | 134.67 |
| 6 | 38.33 | 95.00 |
| 7 | 83.67 | 144.33 |
| 8 | 84.00 | 145.33 |
| 9 | 70.00 | 144.33 |
| 10 | 60.33 | 110.33 |
| 11 | 69.00 | 129.33 |
| 12 | 55.33 | 104.33 |
| 13 | 57.00 | 108.67 |
| 14 | 56.33 | 105.00 |
| 15 | 49.00 | 99.00 |
| 16 | 93.00 | 168.00 |
| 17 | 69.00 | 127.33 |

Table S2: ANOVA values for functional parameters of muffin flour

| **Model parameters** | **WHC** | **OHC** |
| --- | --- | --- |
| **Model** | < 0.0001^*^ | 0.0009^*^ |
| **A-Pea flour** | < 0.0001 | 0.0007 |
| **B-Sugar reduction** | < 0.0001 | 0.0022 |
| **C-Cooking Time** | 0.0002 | 0.0029 |
| **AB** | - | 0.7443 |
| **AC** | - | 0.7012 |
| **BC** | - | 0.3706 |
| **A²** | < 0.0001 | 0.0010 |
| **B²** | 0.0005 | 0.0025 |
| **C²** | < 0.0001 | 0.0005 |
| **R^2^** | 0.9590 | 0.9496 |

*^*^Significant difference*

Table S3: Experimental design using factors for responses of physical parameters of muffins

| **Run** | **Height**  **(cm)** | **Density**  **(g/cm^3^)** | **Baking Loss**  **(%)** | **Volume**  **(cm^3^)** | **Mass**  **(g)** | **Texture**  **(N)** |
| --- | --- | --- | --- | --- | --- | --- |
| 1 | 4.13 | 0.89 | 14.10 | 47.74 | 42.47 | 15.03 |
| 2 | 4.47 | 0.93 | 12.17 | 45.75 | 42.76 | 13.13 |
| 3 | 4.23 | 1.00 | 7.71 | 45.91 | 45.90 | 6.33 |
| 4 | 4.17 | 0.84 | 14.47 | 42.35 | 35.39 | 21.03 |
| 5 | 4.13 | 0.90 | 7.18 | 50.09 | 45.23 | 16.30 |
| 6 | 4.27 | 0.90 | 11.19 | 48.58 | 43.91 | 10.80 |
| 7 | 4.43 | 0.98 | 12.86 | 43.06 | 42.03 | 17.70 |
| 8 | 4.17 | 0.84 | 14.47 | 42.35 | 35.39 | 21.03 |
| 9 | 4.13 | 0.86 | 11.43 | 50.28 | 43.49 | 10.27 |
| 10 | 4.23 | 0.86 | 5.50 | 54.25 | 46.50 | 14.00 |
| 11 | 4.43 | 0.87 | 14.41 | 48.06 | 42.03 | 10.83 |
| 12 | 4.17 | 0.98 | 15.88 | 42.13 | 41.39 | 13.27 |
| 13 | 4.30 | 0.88 | 12.36 | 48.27 | 42.25 | 12.30 |
| 14 | 4.33 | 0.87 | 8.69 | 51.34 | 44.49 | 13.43 |
| 15 | 4.13 | 1.00 | 13.97 | 42.61 | 42.51 | 14.43 |
| 16 | 4.17 | 0.84 | 14.47 | 42.35 | 35.39 | 11.40 |
| 17 | 4.13 | 0.82 | 14.37 | 51.46 | 42.05 | 15.30 |

Table S4: ANOVA values for physical parameters of muffins

| **Model parameters** | **Height** | **Density** | **Baking loss** | **Volume** | **Mass** | **Texture** |
| --- | --- | --- | --- | --- | --- | --- |
| **Model** | 0.3357^NS^ | 0.1550^NS^ | 0.0036^*^ | 0.0178^*^ | 0.0004^*^ | 0.9576^NS^ |
| **A-Pea flour** | 0.8045 | 0.0490 | 0.4248 | 0.0213 | 0.7406 | 0.9536 |
| **B-Sugar**  **reduction** | 0.5399 | 0.2765 | 0.1665 | 0.4499 | 0.3564 | 0.8639 |
| **C-Cooking Time** | 0.8654 | 0.1850 | 0.0120 | 0.0231 | 0.0764 | 0.8212 |
| **AB** | 0.9107 | 0.5098 | 0.0034 | 0.0690 | 0.0419 | 0.9042 |
| **AC** | 0.0714 | 0.2983 | 0.0440 | 0.0736 | 0.2383 | 0.8613 |
| **BC** | 0.5876 | 0.8930 | 0.0009 | 0.1806 | 0.0215 | 0.9105 |
| **A²** | 0.6107 | 0.0551 | 0.2298 | 0.0567 | < 0.0001 | 0.5380 |
| **B²** | 0.3576 | 0.0436 | 0.0068 | 0.0310 | < 0.0001 | 0.1701 |
| **C²** | 0.0556 | 0.4677 | 0.0448 | 0.0043 | < 0.0001 | 0.5127 |
| **R²** | 0.6430 | 0.7392 | 0.9244 | 0.8755 | 0.9608 | 0.2689 |

*^*^Significant difference*

Table S5: Experimental design using factors for responses of sensory evaluation of muffins

| **Run** | **Color** | **Appearance** | **Texture** | **Taste** | **After Taste** | **Overall Acceptability** |
| --- | --- | --- | --- | --- | --- | --- |
| 1 | 6.60 | 6.43 | 6.60 | 6.27 | 6.33 | 6.60 |
| 2 | 5.93 | 5.93 | 6.40 | 6.20 | 6.13 | 6.27 |
| 3 | 5.53 | 5.57 | 5.53 | 5.47 | 5.53 | 5.53 |
| 4 | 5.67 | 5.86 | 5.47 | 6.00 | 6.40 | 6.00 |
| 5 | 5.47 | 5.14 | 5.33 | 5.40 | 5.73 | 5.40 |
| 6 | 6.40 | 6.43 | 6.87 | 7.07 | 7.00 | 6.87 |
| 7 | 5.93 | 5.93 | 6.27 | 5.93 | 5.80 | 5.73 |
| 8 | 5.67 | 5.86 | 5.47 | 6.00 | 6.40 | 6.00 |
| 9 | 6.80 | 7.00 | 6.80 | 6.80 | 6.67 | 6.93 |
| 10 | 6.80 | 6.57 | 6.27 | 6.07 | 6.20 | 6.53 |
| 11 | 6.33 | 6.29 | 6.00 | 6.20 | 6.07 | 6.33 |
| 12 | 5.47 | 5.57 | 5.87 | 5.80 | 5.80 | 5.67 |
| 13 | 7.07 | 7.00 | 6.67 | 6.53 | 6.33 | 6.87 |
| 14 | 5.87 | 5.93 | 6.00 | 6.53 | 6.27 | 6.47 |
| 15 | 5.33 | 5.57 | 5.67 | 5.73 | 5.80 | 5.73 |
| 16 | 5.67 | 5.86 | 5.47 | 6.00 | 6.40 | 6.00 |
| 17 | 6.80 | 6.79 | 6.53 | 6.40 | 6.53 | 6.47 |

Table S6: ANOVA values for sensory parameters of muffins

| Model Parameters | Color | Appearance | Texture | Taste | After taste | Overall acceptability |
| --- | --- | --- | --- | --- | --- | --- |
| Model | 0.0034^*^ | 0.0014 ^*^ | 0.0052 ^*^ | 0.0083 ^*^ | 0.0242 ^*^ | 0.0015 ^*^ |
| A-Pea flour | 0.0021 | 0.0012 | 0.0139 | 0.0753 | 0.1333 | 0.0107 |
| B-Sugar  Reduction | 0.0209 | 0.0111 | 0.0176 | 0.0128 | 0.0341 | 0.0023 |
| C-Cooking Time | 0.2487 | 0.1147 | 0.0825 | 0.0179 | 0.0322 | 0.0148 |
| AB | - | - | - | 0.1431 | - | - |
| AC | - | - | 0.0238 | 0.0445 | - | 0.1037 |
| BC | - | - | - | - | - | - |
| A² | - | - | 0.0057 | - | - | - |
| B² | - | - | 0.0141 | - | 0.1411 | - |
| C² | - | - | 0.0259 | - | - | - |
| R² | 0.6387 | 0.6873 | 0.8411 | 0.7181 | 0.5812 | 0.7456 |

*^*^Significant difference*

Table S7: Point confirmation of functional optimized muffins

| **Analysis** | **Predicted Mean** | **Data Mean** | **95% PI low** | **95% PI high** |
| --- | --- | --- | --- | --- |
| **Height** | 4.16 | 4.43 | 3.86 | 4.47 |
| **Density** | 0.83 | 0.86 | 0.701 | 0.961 |
| **Baking loss** | 6.60 | 8.08 | 3.92 | 9.88 |
| **Volume** | 54.28 | 52.50 | 47.9 | 61.0 |
| **Mass** | 45.03 | 45.10 | 41.9 | 48.3 |
| **Texture** | 14.36 | 5.50 | 3.22 | 32.1 |
| **WHC** | 60.98 | 67.21 | 52.0 | 70.5 |
| **OHC** | 121.99 | 138.40 | 101.0 | 144.0 |
| **Color** | 6.85 | 7.47 | 5.91 | 7.84 |
| **Appearance** | 6.87 | 7.33 | 6.04 | 7.74 |
| **Texture HS** | 6.49 | 7.13 | 5.76 | 7.26 |
| **Taste** | 6.35 | 7.40 | 5.61 | 7.12 |
| **After taste** | 6.63 | 7.40 | 5.94 | 7.36 |
| **Overall acceptability** | 6.77 | 7.60 | 6.04 | 7.53 |
